# Tackling climate change through craft development: The case of rural women in uPhongolo Local Municipality

**DOI:** 10.4102/jamba.v13i1.1140

**Published:** 2021-09-30

**Authors:** Antonia T. Nzama

**Affiliations:** 1 Department of Recreation and Tourism, Faculty of Arts, University of Zululand, KwaDlangezwa, South Africa

**Keywords:** Climate change, natural capital, sustainable livelihoods, innovative entrepreneurship, craft development

## Abstract

Climate change is a global phenomenon that is affecting all humanity. Bearing the harshest brunt of environmental, social and economic shocks are the world’s poorest and those in vulnerable conditions such as women in rural areas. Rural areas have experienced a decline in the dependence on agriculture and livestock farming because of climate change, thus forcing people especially women to look for alternative sources of sustainable livelihoods (SLs). The objective of this study was to establish the extent to which craft development can be used as an alternative livelihood by women in uPhongolo Local Municipality in KwaZulu-Natal to mitigate the effects of climate change. This study adopted a SL theoretical framework to explain how women in the study area used craft development to improve their livelihoods. A survey method was adopted for this study using both qualitative and quantitative approaches. Non-probability sampling strategy using a purposive sampling technique was used to select 50 women crafters from uPhongolo Local Municipality. Face-to-face interviews using questionnaires, which had both closed and open-ended questions, were conducted. These allowed for the collection of numeric data and simultaneously allowed respondents to express themselves and elaborate on the structured questions. The Software Programme for Social Science (SPSS) was used to analyse quantitative data that had been generated using structured interviews and categorised qualitative data. The findings indicated that innovative entrepreneurship using natural capital readily available in the area for craft development and linking the products to the market play a significant role in improving SLs of women in the study area. The study recommends that capacity-building programmes be provided to equip rural women with skills that would enhance their ability to respond to natural hazards such as climate change.

## Introduction

Climate change is a global phenomenon that has resulted in a loss of livelihoods because of declining dependence on agriculture and livestock farming (Aziz 1978; Harris 1982; Kheiri & Nasihatkon 2016; Nawrotzki, Hunter & Dickinson 2012; World Bank 2008). Natural hazards such as flood and drought, which are a result of climate change, dictate that alternative livelihood options be explored to either substitute or supplement agriculture and livestock farming (Shackleton & Shackleton 2004). Climate change and other factors such as change in lifestyles, urbanisation, brain drain and sudden changes in the economic, social or bio-physical environments have led to increased levels of poverty and loss of livelihoods particularly in rural areas (Mbaiwa 2004; Nzama 2008; Shackleton & Shackleton 2004).

The most vulnerable to the impact of climate change are the world’s poorest, marginalised segments of the society who are largely women and those in vulnerable conditions such as rural areas, which are generally characterised by deep levels of poverty (Alam, Bhatia & Mawby 2015; Kelman 2013; Nyawo & Mashawu 2019). Women, who, because of the socially and culturally constructed roles, ascribed to them, are on the frontline as caregivers for their families and communities are negatively affected by climate change (Dugan 2019; Nhemachena et al. 2014). Although women are seen as victims of climate change, they are also perceived as powerful change agents, because of their strength and ability to identify resources that have a potential for saving climate change-affected communities and come up with practical solutions (Resurreccion 2013). This means despite women being disproportionately affected by climate change, they play a crucial role in finding ways to adapt and mitigate and even identify opportunities for reducing vulnerability to climate change (Dankelman 2010; Glazebrook 2011; Habtezion 2013; Kelman 2013). Although studies confirm that women’s knowledge and skills to adapt their efforts towards mitigation of the negative effects of climate change contribute to their ability to provide alternative livelihoods for their families, there is dearth of information on how they can use natural resources that are readily available in the areas for craft development as a strategy to mitigate the negative impacts of climate change. The purpose of this study therefore was to establish the extent to which craft development can be used as an alternative livelihood by women in uPhongolo Municipality in Northern KwaZulu-Natal to mitigate the effects of climate change.

### Problem statement

Climate change has affected the entire humanity, but its effects are experienced differently at various regional and local levels and have subsequently been touted as the biggest development challenge that faces humanity. Climate change has resulted in an evident decline of productivity in crop and livestock farming and a loss of livelihoods that are dependent on climate variability (Habtezion 2013; Kelman 2013). Rural communities in particular have lost their agricultural productivity and have witnessed increased poverty. Rural households in general and as it is the case with women in uPhongolo Local Municipality find themselves forced to engage in diverse types of income-generating livelihood activities (Dorward et al. 2001).

The main source of livelihood for rural communities in Northern Zululand where the study area is located, have from time immemorial depended on crop and livestock farming as their main source of livelihood. Most households in these communities are headed by women. Unfortunately, in recent years these communities have experienced a decline in crop and livestock farming because of climate change. This decline has made these communities vulnerable and calls for re-thinking of other livelihood strategies. With continuing devastating effects of climate change, these communities find themselves forced to find strategies of adapting to the negative impacts of climate change and to engage in diverse types of income generating livelihood activities to either substitute or supplement the original livelihoods (Dorward et al. 2001).

One of the strategies of adapting to the negative impacts of climate change is to find an alternative to heavy reliance on agriculture as a primary source of livelihood, shifting from heavy reliance on traditional livelihoods that are based on agriculture and livestock or finding alternative livelihoods to supplement or substitute basic livelihoods (Denton 2010). Studies have indicated that craft development can trigger the transformation of traditional livelihood whenever the need arises (Lasso & Dahles 2018). Craft development has remained a popular activity over the years as a means of making financial gains (Malema & Naidoo 2017). Studies have not focused on the extent to which women can use natural resources for craft development as a strategy for providing alternative livelihoods as a response to the devastating impacts of natural hazards such as climate change. The purpose of this study was to establish the extent to which craft development can be used by women in uPhongolo Local Municipality as an alternative livelihood in order to mitigate the effects of climate change.

This article uses the concept of sustainable livelihoods (SLs) to assess the role that craft development can play in enhancing the ability of women in the study area to use craft making as a strategy to mitigate the negative economic impacts of climate change.

### The study area

**FIGURE 1:**
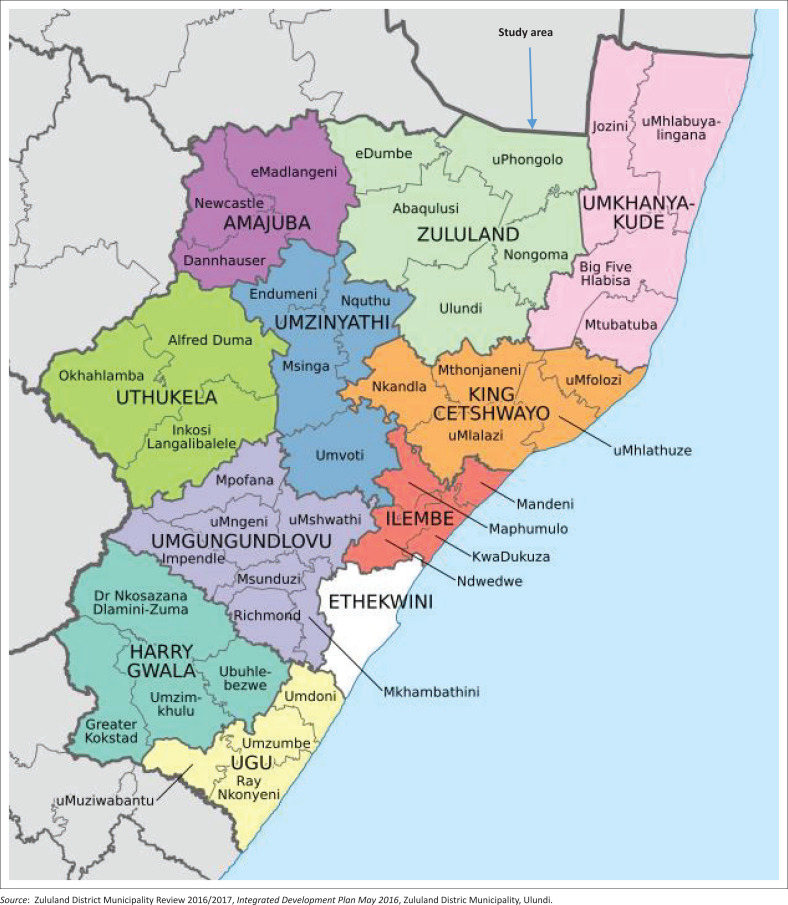
Map of the study area: uPhongolo local municipality (one of the five local municipalities with the Zululand District Municipality).

This study was conducted at uPhongolo Local Municipality, which is one of the five local municipalities within the Zululand District Municipality in Northern KwaZulu-Natal in South Africa. The study area is reflected in Figure 1. It is the third largest local municipality with a high number of households that are located in rural areas (81%) and also a high percentage (41%) of unemployment. This situation justifies the necessity of implementing projects that would address social challenges such as poverty and unemployment especially for people that reside in rural areas.

### Theoretical framework of the study

The SL theoretical framework forms the basis for this study because of its relevance in supporting an integrative way of thinking about strategies for poverty reduction and also in highlighting ways of supplementing livelihoods for survival (Chambers 1992; Petersen & Pedersen 2010; Sati & Prasad 2017; Shen, Hughey & Simmons 2008). In the face of climate change resulting in the loss of livelihoods and increase in poverty levels particularly in rural areas, there is a need for strategies to turn the situation around (Nyawo & Mashawu 2019). If poverty reduction is to be taken seriously, it cannot be left in the hands of the women or those communities that are feeling the brunt of climate change, but a multi-stakeholder approach has to be adopted. Multi-stakeholder approaches can align opportunities for livelihood activities at local levels with policy development. These approaches may also enhance the creation of a conducive and an enabling environment for establishment of alternative livelihood activities. Ashley and Carney (1999) asserted that macro-level structures and processes support people to build on their strengths to sustain their households.

The relevance of the SL framework for this study is also because of its focus not only on factors that affect the livelihoods of the poor rural people but also on those who make them vulnerable such as climate change, the assets and resources that enable them to thrive and survive, and the policies and institutions that impact their livelihoods. Also of importance is the ability of this framework to display how SLs are achieved through access to a range of livelihood assets (natural, economic, human, financial and social capital), which are combined in the pursuit of different livelihood strategies (Kabir et al. 2012; Mbaiwa 2004). Clearly, this framework makes it possible to connect people and the overall enabling environment that influences the outcomes of livelihood strategies (Serrat 2008).

Chambers and Conway (1992) defined a livelihood as capabilities, assets and activities required for a means of living and put emphasis on the sustainability of the livelihoods. Krantz (2001) asserted that of the various components of a livelihood, the most complex is the portfolio of assets out of which people construct their living, which includes both tangible and intangible assets such as claims and access. A livelihood can not be seen in isolation, instead, it should be viewed as connected by environmental, economic, political and cultural process to wider national, regional and global agendas, which seeks to fulfil both material and experiential needs (Kabir et al. 2012). A livelihood is sustainable if it can cope with and recover from stress and shocks, maintain or enhance its capabilities and assets, and provide SL opportunities for the next generation and contribute net benefits to other livelihoods at the local and global levels and in the long and short term (Chambers & Conway 1992). Kabir et al. (2012) pointed out that a livelihood is environmentally sustainable when it maintains or enhances the local and global assets on which livelihoods depend and has net beneficial effects on other livelihoods. The SL approach therefore highlights the ability of the community to enhance its assets and capabilities to respond to the shocks and stresses, such as climate change, over time (Norton & Foster 2001).

Even though the SL approach is widely used in poverty and rural development research, there is no universal agreement about its precise emergence and definition as individuals, organisations, governments and development practitioners have based the definition on their own interpretation and understanding (Shen et al. 2008). Another contributory factor is that this approach has evolved within the context where practitioners continuously seek to maximise the effectiveness of their interventions to help people improve their livelihoods (Krantz 2001; Morse & McNamara 2013).

Regarding its emergence, some studies indicate that the SL approach was proposed in the 1980s, whilst others claim that it came to prominence in the UK Department for International Development as a follow-up process to the White Paper on International Development of 1997 (Ashley 2002; Cahn 2002; Carney 1999; Chambers & Conway 1992; Ellis 2000; Morse, McNamara & Acholo 2009; Shen 2009). According to Krantz (2001), the SL idea was first introduced by the Brundtland Commission on Environment and Development, and the 1992 United Nations Conference on Environment and Development expanded the concept, advocating for the achievement of SLs as a broad goal for poverty eradication. What is evident in numerous studies is that the SL approach has since the 1990s become the dominant approach to implement and facilitate development interventions by a number of major international agencies (Fujun et al. 2008; McNamara & Acholo 2009; Kabir 2012; Bromley 1990; Chambers & Conway 1991; DFID 2000; Mahmood et al. 2016; McKulka 2009; Tao & Wall 2009; Swanborn 2010).

As an approach, the SL is seen as an organising framework that addresses the dynamic dimensions of poverty and well-being of communities through establishing a typology of assets, which individuals, households and communities utilise to maintain livelihoods under changing conditions (Cahn 2002). The main distinguishing feature of this approach is that it analyses livelihoods within a comprehensive framework and offers information for policymaking at local, national and global levels and it also emphasises a holistic and integrated thinking about poverty reduction and rural development (Chambers 1992; Chambers & Conway 1992; Morse & McNamara 2013; Norton & Foster 2001).

One of the advantages of the SL approach is that it provides a wider and better understanding of the opportunities for development activities and their likely impact whilst placing people and their priorities at the centre of analysis. Morse and McNamara (2013) also added that its application is flexible and adaptable to specific local settings. The other value of the SL approach is that it is guided by core principles, which can be integrated in any rural development setting. According to Ashley and Carney (1999), these principles include the following: people centredness, responsiveness, participatory in nature, working at multi-levels, which include public and private sector partnerships, it upholds sustainability principles and offers dynamic livelihood strategies.

Multi-stakeholder approach can link various sectors that would strengthen the possibility of making alternative livelihoods a reality. As an example, craft development can be linked to the tourism sector, which is regarded as the biggest and fastest growing industry in the world and as a vehicle for poverty alleviation and a bridge between the poor and the affluent (Goodwin 2002, 2006; Holland, Dixey & Burian 2003; Jamieson, Goodwin & Edmunds 2004; Shen 2009; Zhao & Ritchie 2007; Zhou 2014). With the recognition of the potential of tourism and its comparative advantages in reducing poverty, especially in rural areas, it is increasingly perceived as a livelihood strategy particularly in rural areas (Goodwin 2002). Policymakers have also come to support the view that tourism has a potential to reduce poverty in rural areas and contribute to development in general (Goodwin 2002, 2006; UNWTO 2002). In 2002, the UNWTO launched ‘Tourism and Poverty Alleviation’ at the World Summit on Sustainable Tourism in Johannesburg, which re-examined the role of tourism in reducing poverty, and a conclusion was drawn that tourism could be one of the few effective means of contributing to poverty alleviation in rural areas if properly managed.

To contain the cost of craft development, readily available assets such as the natural and human capital were utilized for craft development.. One of the key features of the SL approach that relate to this study is that it highlights five forms of capitals or assets that are fundamental to the poor, which include natural, social, human, physical and financial capitals (Carney 1999; Daskon & MacGregor 2012; Shen et al. 2008). Of these capitals, the study focused on natural and human because these were readily available in the area. Natural capital is the term used for the natural stocks from which flows resources and services that are useful for livelihoods (Kabir 2012). Natural capital includes the world’s stocks of natural assets such as fauna, flora, geology, soil, air and water all of which sustain livelihoods. In its broad sense, natural *capital* covers tangible resources such as trees and land and more intangible products such as the atmosphere and knowledge.

Also of importance is the extent to which the assets are accessible. Central to the SL approach is not only the availability but also access to various capital assets and strategies that provide livelihood options for rural households and the markets, which serve as outlets for the products (Carney 1999; Nawrotzki, et al. 2012; Serrat 2008; Zhou 2014). As an example, access to the raw material that would be required for craft production and the knowledge of how products with a competitive edge would be designed and developed for the tourism sector is significant. Carney (1999) pointed to the significance of access to land, water and wildlife from which households engage in pursuits of livelihood activities for both sustenance and income generation. Access to natural capital can facilitate acquisition of other livelihood assets such as financial capital, for example, income generation through products woven with locally collected reeds (Pereira, Shackleton & Shackleton 2006). Natural resources can act as a safety net to which households turn to whenever disaster such as harvest failure, natural hazards, climate and death of a breadwinner strike (Nawrotzki et al. 2012).

Some studies indicate that the natural resources are not only central to livelihoods in rural areas but they are also associated with higher levels of financial, human and social capital. One of the cornerstones of the success of the project that forms the basis of this study was the identification of the assets that were readily available within the community. To provide any meaningful intervention to poverty reduction using the SL approach, it is important to examine the types of assets that are available and align these with the different livelihood strategies (Dorward et al. 2001). Livelihood strategies are the activities that are utilised to generate the means of household survival, which in the case of this study would be craft development. These livelihood strategies can be classified as natural resource-based and non-natural resource-based (Carney 1999; Ellis 2000).

## Research methodology

The study was conducted over a period of 1 year from January to December 2018 based on a project that was designed such that natural resources that were readily available in the area would be harvested and used for craft development. This approach was used in order to reduce business start-up costs. After conducting a scoping exercise, it was established that the natural resources that could be found everywhere in the area and which were not affected by the effects of climate change were the soapstone and different types of wood from the natural forest. Of the two types of natural resources, women opted for the soapstone. To ensure the sustainability of the project, these women were taught both practical and entrepreneurial skills, which included, amongst others, the skill of designing products, translating mental images to the paper to facilitate replication in case there are orders of a specific shape and size; the significance of producing attractive and unique designs to create a demand, pricing and packaging, to always match supply with demand; and strategies of mass production.

To make sure that the objectives of the study were achieved, a mixed method approach was used. This approach is appropriate for this study because it allowed the researcher to focus the investigation on the dynamics of a group of participants in the project (Welman, Kruger & Mitchell 2012). Quantitative and qualitative approaches were used to collect data that were required to address the objectives of the study. The reason for choosing mixed method approach was that quantitative and qualitative approaches complement each other and therefore allowed for more complete analysis of the study and also provided an in-depth understanding of some trends and patterns and relationships between variables (Brotherton 1999; ed. Maree 2012).

The use of the quantitative and qualitative approaches was also seen to be appropriate because it gave the respondents an opportunity to explain or elaborate on quantitative and qualitative data. In this study, not only did the mixed method approach allow for the description and understanding of the activities, actions, behaviour and practices of the crafters who participated in the project from their own perspective but it also allowed the flexibility in data collection and data analysis strategies (Stake 2003). Babbie and Mouton (2001:272) argued that it is only ‘if one understands events against the background of the whole context and how such context confers meaning to the event than one can truly claim to understand the events’. In line with the mixed method approach, the questionnaires were used as the research instrument. These questionnaires had both closed-ended questions that allowed for the collection of numeric data and open-ended questions that allowed respondents to express themselves and elaborate on the structured question. The research process therefore allowed for both structured and unstructured interviews to be used to collect data.

Non-probability sampling was seen to be appropriate for this study because respondents were selected purposively based on their skills in craft development and their availability to participate in the craft development project (Clark et al. 1998). The population for this study included all known crafters within uPhongolo District. A purposive sampling strategy was used to select 50 participants on the basis of their experience in craft development and availability to attend capacity-building sessions for a year where they would be trained in practical and entrepreneurship skills. The expected outcome of the project was that at the end of the project women’s rudimentary skills in craft development would be refined so that they are able to produce craft products of a high quality with unique designs with a potential of entering the tourism market.

Secondary data collection included a review of literature on sustainable rural livelihoods, SLs and craft development for tourism.

Inductive analysis of data was adopted in an attempt to examine the research findings as they emerged from the frequent, dominant or significant themes inherent in the raw data. Inductive codes had been developed through the direct examination of raw data. Related codes were combined and placed into various categories. SPSS was used to analyse quantitative data that had been generated using structured interviews and categorised qualitative data. Through the use of SPSS tables and frequency tables were generated from the data that were collected. The descriptive statistics generated through SPSS provided the socio-economic profile of the respondents. Data interpretation was carried out to seek emerging patterns and explanation of data so as to draw conclusions.

### Ethical considerations

The Research Ethics Committee of the University of Zululand approved the research methodology as contained in the proposal of the study and gave the Ethical Clearance Certificate to the author to proceed with the implementation of the study, reference number: UZREC171110-030.

## Findings

The demographic characteristics of respondents are presented in Table 1.

**TABLE 1:** Demographic characteristics of respondents (*n* = 50).

Profile	Variable	Frequency	%
Gender	Female	50	100
Age	15–24	8	16
	25–39	22	44
	40–59	16	32
	60 and above	4	8
Level of education	No formal education	4	8
	Primary education	24	48
	Secondary school	16	32
	High school (matric)	6	12
	University	0	0
Income per month	R0 – R1000	22	44
	R 1000 – R200 000	14	28
	R2001 – R 300 000	8	16
	R3001 – R 400 000	6	12
	R4001 – R5000	0	0
	R 5001 and above	0	0
Number of members in the household	1–4	4	8
	5–8	34	68
	9–12	11	22
	More than 13	1	2
Are you the breadwinner	Yes	21	42
	No	29	58
Source of income	Formal employment	0	0
	Part-time jobs	12	24
	Government grant	22	44
	Sale of craft	6	12
	Crop farming	4	8
	Livestock farming	4	8
	Combination of crop and livestock farming	2	4
Period of stay in current residence	Less than 5 years	2	4
	5–10 years	6	12
	11 and over	42	84

The findings of the study indicate that all crafters who joined the craft development project were females. These women were mainly heads of their families as the majority of males move to urban areas to seek job opportunities (2016/2017 Integrated Development Plan). The findings also indicated that the level of education of these women was low with the majority having passed only primary education (48%). This low level of education contributed to a high unemployment rate. None of the participants had a full-time job and the majority of them (44%) earned not more than R1000 per month, which is inadequate to support their families. The findings also indicated that the majority of them were between the 25 and 39 years old (44%), which is an indication of joblessness amongst young people. What is worth mentioning is that 42% of these women were breadwinners. The Zululand District Municipality Review 2017/2018 supports these findings and indicates that the Zululand District Municipality has one of the highest dependency ratios, which is a crude measure of poverty, and this demonstrates a need to sustain social development programmes and support to local communities.

Such high unemployment points to a need for these women to find ways of getting income for the survival of their families. Respondents were asked to indicate their sources of livelihood, which would reflect economic activities they participated in before they joined the craft development project. The findings indicated that they relied on diverse sources of income, which were clustered in the following categories: part-time jobs (12%), government grant (44%), sale of craft (24%), crop farming (8%), livestock (8%) and a combination of crop and livestock farming (2%). Findings indicated a clear decrease in the reliance on crop and livestock farming. One respondent mentioned that:

‘We can no longer rely on agriculture because climate change has resulted in crop fields that are uncultivated and the grazing lands that have become bare because of the loss of grass to feed the livestock.’ (56 year old, female, unemployed)

The other respondent also indicated that:

‘[*E*]ven though we knew we needed to do something else to substitute the livelihoods obtained through crop and livestock farming we did not have money and the skills to start other ventures.’ (40 year old, female, breadwinner)

The natural resources that had been made available through the scoping process of the project were the soapstone and wood. Of the two natural resources, soapstone was chosen by all women. Respondents were asked if they had used soapstone for craft development before. All respondents indicated that they had never carved the soapstone before but were willing and determined to learn. One of them mentioned that ‘As a born crafter I can use any resource to develop any type of craft if I am given an opportunity’. Some of them (58%) believed that carving soapstone will increase their chances of creating unique products because the market is flooded with craft items that are made out of grass, which is what they had been producing all along. One respondent mentioned that:

‘I have never gone to school but I learned a skill of craft development from my parents who also learned from their parents – it is a skill that has been handed down through generations.’ (female, unemployed, no formal education)

All respondents also indicated that they had never used any design or pattern to develop the products but expressed their readiness to learn how to design and package products for the market. Their enthusiasm to learn was a clear move towards commercialisation of their products instead of producing items for subsistence purposes.

Crafters were asked if they were willing to form a business cooperative 78% indicated that they have always worked as individuals and/or families and only 22% were at some stage members of cooperatives, which have since disintegrated. At that stage, all crafters expressed their willingness to become members of a cooperative. One of the respondents mentioned that they ‘have always wanted to expand their craft production to a level where they can form a business’ but did not know how. To satisfy their desire to become entrepreneurs, crafters were assisted to form a business cooperative and to get it registered. At the end of the training programme, some crafters (67%) saw themselves as entrepreneurs who collectively produced different products with unique shapes and had become members of a fully registered cooperative with a bank account. A workshop on leadership and corporate governance provided a foundation for their entry into small business sector and turned them into entrepreneurs.

They were asked to indicate their understanding of ‘entrepreneurship’ and ‘entrepreneurial innovation’. A range of responses were provided, which included ‘owning a business (95%), starting your own business (67%), producing items for a specific market (64%), producing a unique product (63%) and coming up with a new idea (64%)’. They were asked to indicate what they learned and which task they can confidently do on their own without the assistance of the trainers. Their responses are summarised in Table 2.

**TABLE 2:** Skills learned through the training programme.

Skills	%
Drawing a design of the product	100
Designing different shapes of products	89
Identification of quality of the stone/wood	87
Cutting the stone/wood to the desired size	89
Drawing to scale (small, medium, large)	77
Pricing of the products	68
Business management	65
Budgeting	62
Financial management	61
Time management	57
Team work	56

Participants also indicated that they believed that income generated from the sale of craft would increase if they focused all their energies on craft production. A majority of them (67%) had stopped participating in other activities in order to focus on the new ‘business’. They also indicated that the flow of income from their business had made their lives better and improved their socio-economic conditions. When asked what they thought might be a challenge in future. The majority (76%) of the respondents indicated that (1) a major threat was a lack of a strong link between their cooperative and the markets to ensure a continuous flow of income, (2) a possibility of the cooperative disintegration and (3) the dependence on the local government support for supply of the soapstone, which had to be collected from the mountain to the community centre where the training was conducted and the supply of the equipment to produce different types of products made of the soapstone. A tractor was required to fetch the soapstone, which is available as huge rocks at the mountain and other parts of the study area. The findings clearly indicated that capacity building and innovative entrepreneurship using natural capital that is readily available in the area for craft development and linking the products to the market can play a significant role in improving SLs of women in the study area.

## Discussion

The key findings of this study clearly indicated that women in the study area can indeed use natural resources for craft development to mitigate the effects of climate change and also to contribute to their livelihoods. Women’s determination and resilience in the coal face of climate change led to the birth of cooperatives and entrepreneurial spirit. Entrepreneurship as it became evident in this study involves the process of shifting ideas into commercial opportunities for value creation (Leach & Melicher 2009). This confirms studies that highlight the ability of women to find ways to identify opportunities for reducing vulnerability to climate change (Dankelman 2010; Glazebrook 2011; Habtezion 2013; Kelman 2013).

These women were prepared to give up using grass and start using soapstone to produce unique and innovative products. Not only did the women have the strength but also the ability to identify resources that can be used to save climate change-affected communities and also come up with practical solutions (Resurreccion 2013). In this case, the solution for women was innovative entrepreneurship using soapstone for craft development. Linking entrepreneurship with innovation, an innovative entrepreneur represents an individual who assembles resources, labour, materials and other forms of assets to productive use for value and proposes valuable changes and innovative ideas in order to earn a living (Baker & Nelson 2005). Entrepreneurs use untapped or underused resources such as the soapstone in the case of the study area, to create new products, try out solutions to counteract the limitations of the local environment and learn by trial and error (Di Domenico, Haugh & Tracey 2010). Women in the study area proved that rural entrepreneurship can happen in areas that are economically depressed with inadequate infrastructure, economic stagnation, low educational levels, unskilled workers and a culture that is generally not supportive of entrepreneurship (Kulawczuk 1998).

Rural entrepreneurship development has been promoted as a vital and effective component of livelihood development, productivity and growth with an ability to reduce poverty and vulnerabilities of rural communities (Ozgen & Minsky 2007). Vulnerabilities are characterised by insecurities projected by the well-being of individuals, households and communities in the face of changes in their external environment. In the case of this study, vulnerabilities were brought by the effects of climate change. Vulnerabilities can be distinguished by internal and external shocks. Shocks such as climate change represent the external side, whilst the feelings of defencelessness and helplessness represent internal facets of vulnerabilities that are caused by the lack of ability and means to cope with these shocks (Serrat 2008).

Even though soapstone was readily available in the study area, studies indicate that availability and access to natural resources alone are not enough to sustain household livelihoods. Strategies have to be found that might lead households to pioneer new products and services (Krokkranikal & Morrison 2011). In this case, entrepreneurship in craft development and targeting tourism as a sector would be an outlet for the products that are developed by these women. Tourism provides many opportunities for entrepreneurs in its formal and informal sectors.

Entrepreneurship is critical in particular where the focus is on craft development for the generation of alternative livelihood. Entrepreneurship would allow for the creation of new and unique products and services, whilst innovation would enable them to implement their creative ideas (Krokkranikal & Morrison 2011; Shane 2003). In the case of this study, it became evident that the women needed entrepreneurial skills to produce and get new products to a very competitive tourism market. Entrepreneurial skills affect the propensity of individuals to become entrepreneurs and the likelihood of their success in sustaining their livelihoods (Gibb 2009; Guta, Vhudzi & Chazovachii 2017). It also became clear that the issue of business and entrepreneurship skills and competencies require suitable education programmes to help develop entrepreneurial mindset of community members and that innovative entrepreneurship can play an important role in contributing to economic growth, job creation and poverty reduction and can help in sustaining and promoting livelihoods for households in the study area. Entrepreneurial capabilities play a critical role in market entry and in the success of new ventures. These capabilities also contribute to the ability of individuals to determine and identify opportunities, run new businesses, drive innovations and learn from and adapt to changing circumstances (Gibb 2009; Minniti & Byrgave 2001).

To ensure the sustainability of the project, these women were taught both practical and entrepreneurial skills, which included, amongst others, the skill of designing products, translating mental images to the paper to facilitate replication in case there are orders of a specific shape and size; the significance of producing attractive and unique designs to create a demand, pricing and packaging, to always match supply with demand; and strategies of mass production. The findings indicated that innovative entrepreneurship using natural capital that is readily available in the area for craft development and linking the products to the market play a significant role in improving SLs of women in the study area.

Based on the findings of the study, it is recommended that capacity-building programmes be provided in order to equip women with skills that enhance their ability to mitigate the effect of climate change which in the case of.

Capacity building programmes are key to equipping rural women with skills that would enhance their ability to respond to vulnerabilities and natural hazards such as climate change. In the case of this study, response included identifying natural capital at their disposal to develop craft products with a competitive edge to enter the tourism industry. There is a need for more studies that would focus on how women can contribute to climate change mitigation and adaptation strategies. Also of importance are studies that would pay attention to strategies of linking women’s cooperatives with markets to ensure sustainability of their cooperatives and continuous flow of income.

## Conclusion

The conclusion presented here is drawn from the study and is informed by the objectives and findings of the study. The objective of this study was to establish the extent to which craft development can be used as an alternative livelihood by women in the study area to mitigate the effects of climate change, and it can be concluded that indeed natural assets have the likelihood of providing a safety net for communities at times when their original livelihoods are declining. The study has indicated how women in the study area responded to the detrimental effects of climate change by embarking on craft development. Finally, it can also be concluded that capacity-building programmes have a high probability of giving people the confidence to find alternative ways of surviving negative impacts of *natural hazards* that result from shocks such as climate change. Capacity-building programmes equip rural people with entrepreneurial mindset and skills, which contribute to their ability to sustain their livelihoods. Climate change is a reality that will continue to affect and force people especially those living in rural areas to find alternative sources of livelihoods.
